# A case report on the management of neglected and forgotten DJ stent for 18 years with severe encrustation and giant bladder stone

**DOI:** 10.1093/jscr/rjae425

**Published:** 2024-06-24

**Authors:** Tsiyon Nigusie Alemu, Habtamu Aderaw, Yacob Sheiferawe, Kinfe Tsehaye

**Affiliations:** Department of Surgery, Urology Unit, School of Medicine, Addis Ababa University, Addis Ababa, Ethiopia; Department of Surgery, Urology Unit, School of Medicine, Addis Ababa University, Addis Ababa, Ethiopia; Department of Surgery, Urology Unit, School of Medicine, Addis Ababa University, Addis Ababa, Ethiopia; Department of Surgery, Urology Unit, School of Medicine, Addis Ababa University, Addis Ababa, Ethiopia

**Keywords:** FECal stent, encrustation, DJ stent, cystolithotomy

## Abstract

Ureteral stents play a vital role in urologic surgeries, aiding in urinary flow maintenance, obstruction alleviation and facilitating healing. However, when stents are forgotten, they can lead to encrustation, resulting in significant patient morbidity and posing challenges for urologists Stent-related complications have been shown to increase with the duration of time the stent is left in place. This report details the clinical presentation, diagnostic process, and treatment of a 68-year-old male patient had a neglected stent placed 18 years ago after extracorporeal shockwave lithotripsy. He presented with severe stent encrustation, a solitary giant bladder stone, and renal stones. The patient underwent a cystolithotomy to remove the bladder stone followed by an ultrasound-guided percutaneous nephrolithotomy with pneumatic lithotripsy in a separate procedure. Preventive strategies such as implementing a stent registry, enhancing patient education, and encouraging follow-up appointments are crucial to avoid such complications.

## Introduction

Ureteral stents play a crucial role in urological surgeries by relieving ureteral obstructions, dilating ureters for easier instrumentation, preventing post-manipulation obstructions, and aiding in healing [1]. It is also used as permanent and therapeutic interventions for pelvic malignant conditions [2]. However, ureteral stents can lead to complications such as encrustation, urinary tract infections, misplacement, and migration, especially if they are forgotten for an extended period of time. Severe encrustation can make simple endoscopic removal challenging, often requiring advanced interventions such as surgical removal.

The management of encrusted ureteral stents depends on the severity of stone formations and encrustation [3].

In this case report, a combination of percutaneous nephrolithotomy (PCNL) and open surgery was utilized to successfully remove heavily encrusted DJ stent. The patient underwent PCNL to address intra-renal stones encrusted at the proximal end of the stent, and cystolithotomy was performed to remove a giant bladder stone.

## Presentation of case

A 68-year-old male, military personnel, presented to our outpatient clinic complaining of persistent right flank pain for the past 2 years alongside worsening lower urinary tract symptoms. He had a history of a double J stent insertion during extracorporeal shock wave lithotripsy performed 18 years ago for a right renal stone. Due to his military duties and limited health knowledge, he had been lost to follow-up.

**Figure 1:**
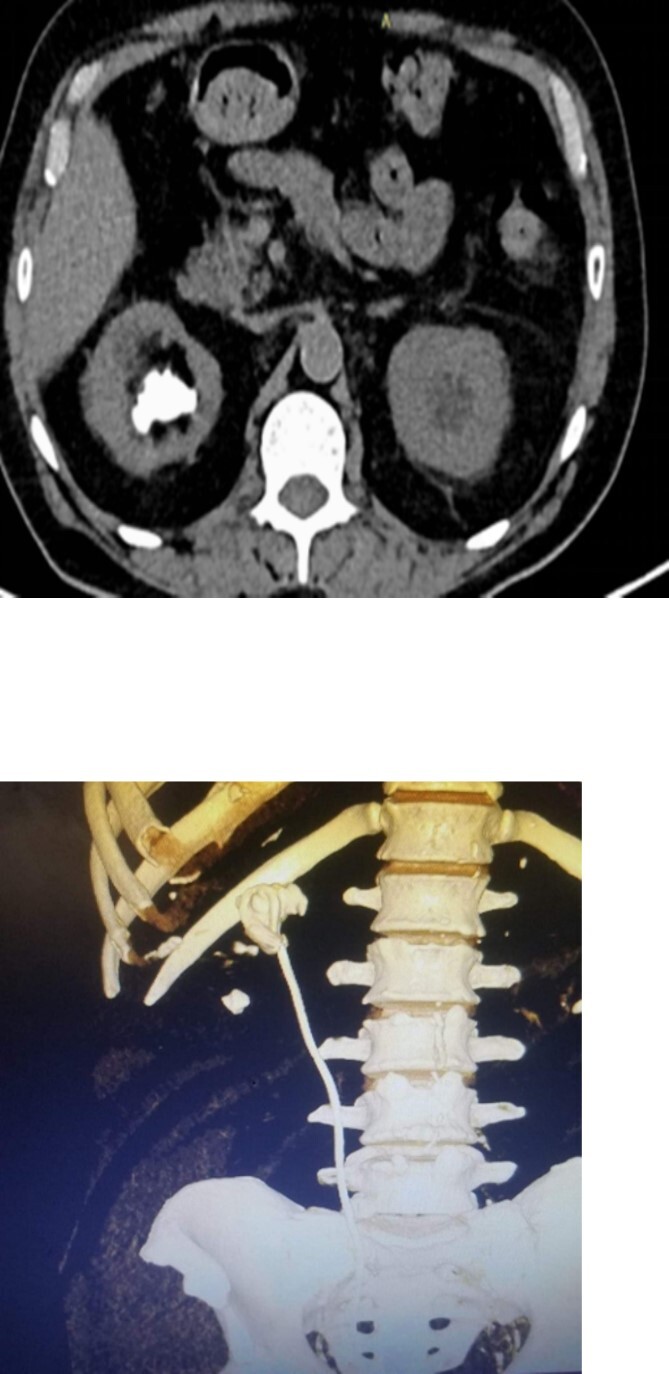
Non-contrast CT scan showed intra-renal stone with significant proximal pigtail calcification and mild degree of hydronephrosis.

**Figure 2:**
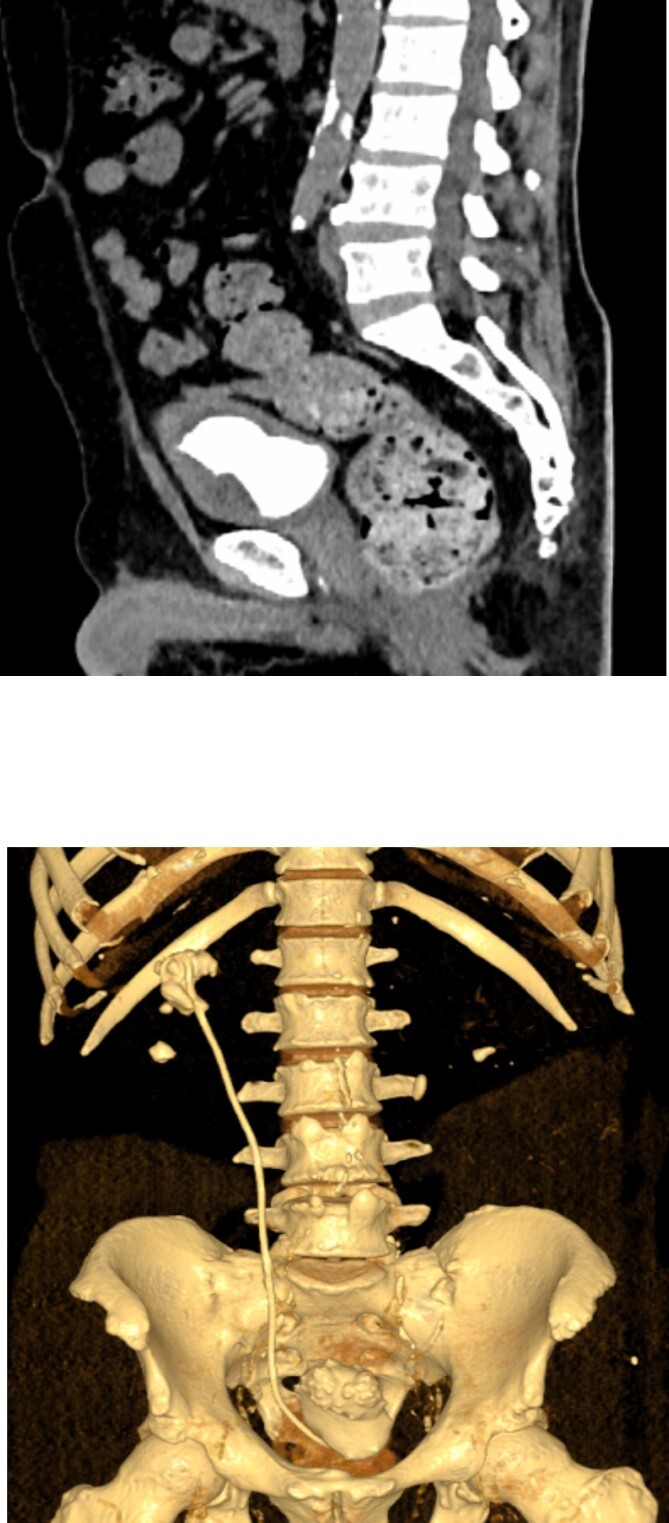
Non-contrast CT scan showed giant bladder stone profoundly embedded in the distal pigtail of the DJ stent.

On physical examination, he had normal vital signs, no fever, and exhibited mild tenderness in the suprapubic area and right costovertebral angle. The results of the laboratory tests were normal, and the serum creatinine level was 0.8 mg/dl. Urine culture was negative for bacteria. Preoperative abdominal and pelvic ultrasound revealed two right renal stones, one measuring 4 cm in the upper pole and 2 cm in the lower pole with mild hydronephrosis, along with a large bladder stone measuring 6 cm, and a shadow of a double J stent. A computed tomography (CT) scan confirmed the presence of a 4 cm upper pole renal stone, a 1.8 cm lower pole renal stone, significant proximal pigtail calcification with mild hydronephrosis (Fig. 1A and B), and a 6 cm bladder stone deeply embedded in the distal pigtail (Fig. 2A and B). Additionally, small intrarenal stones were observed on the left side, along with a proximal ureteric stone measuring 8 mm by 9 mm with mild hydronephrosis.

**Figure 3:**
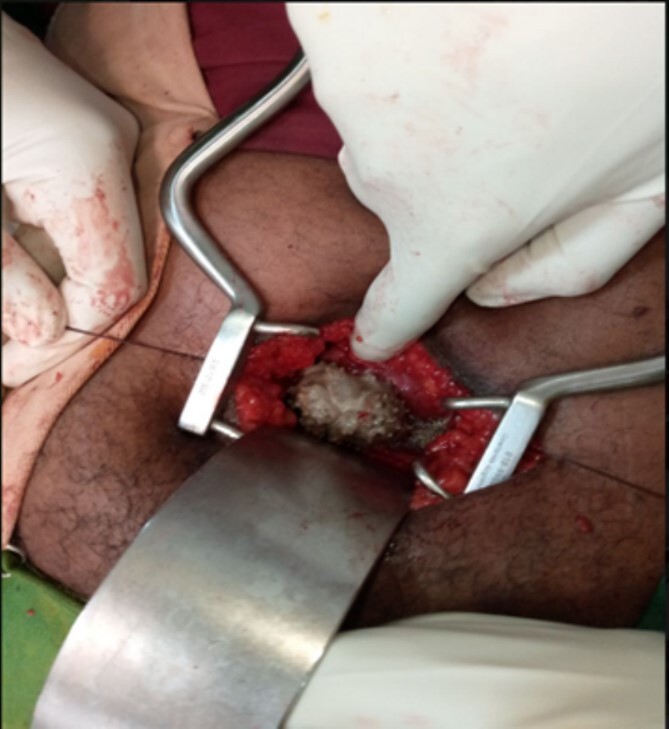
(A) An intraoperative view of the bladder stone as it was seen after the bladder opened anteriorly. (B) The appearance of the bladder stone after removal with the distal segment of the DJ stent.

**Figure 4:**
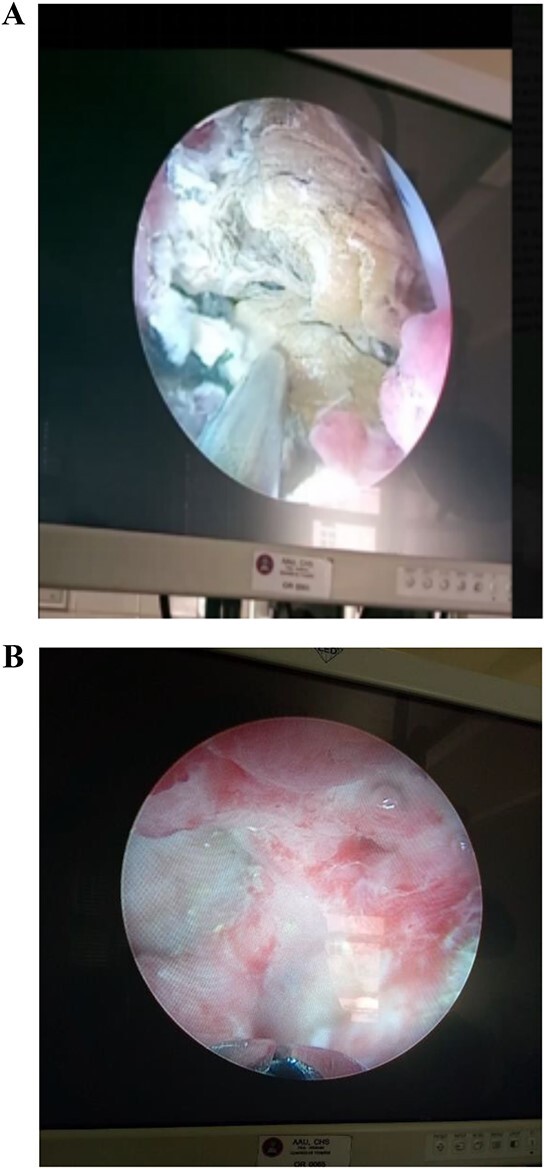
(A) An intraoperative view showing the intra-renal stone being fragmented by pneumatic lithotripsy. (B) Nephroscopic view of calyceal system cleared of the stones.

**Figure 5:**
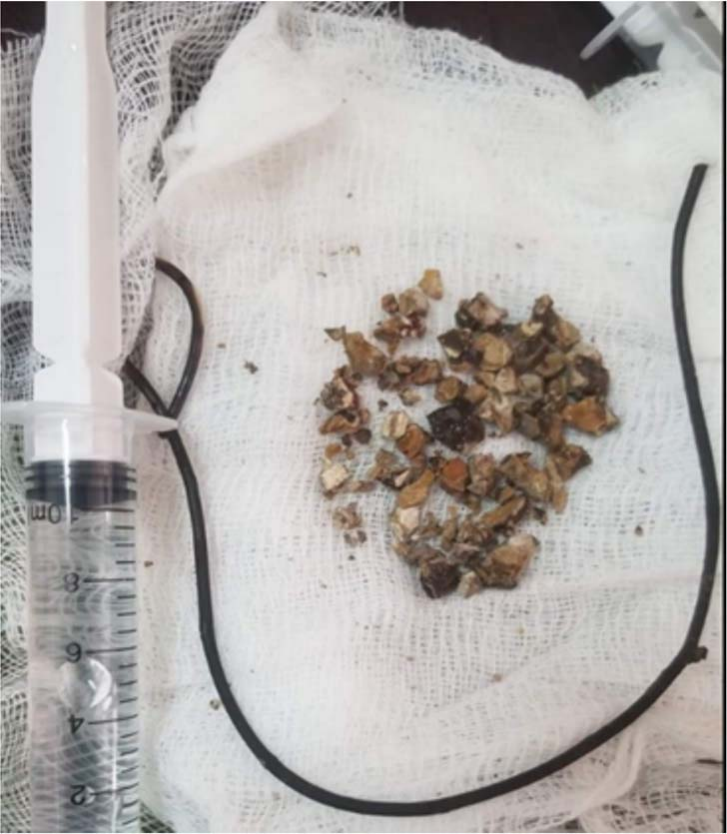
Post-operative appearance of the removed encrusted and the intra-renal stone fragments.

The patient underwent a multi-modal therapy approach involving endourologic treatment and open surgery. Initially, an open cystolithotomy was performed via a Pfannenstiel skin incision with extra-peritoneal approach to remove the 6 ^*^ 5 ^*^ 5 cm bladder stone and the distal part of the encrusted DJ stent which was cut at the ureteric orifice (Fig. 3A and B). Left side semirigid ureteroscopy was also done to remove the ureteric stone. Following discharge, the patient was readmitted after 4 weeks for management of the kidney stones and encrusted DJ stent. Ultrasound-guided percutaneous nephrolithotomy was carried out successfully to remove the kidney stones and the encrusted stent. The Ultrasound-guided percutaneous nephrolithotomy performed over 90 minutes course using a standard nephroscope 24fr. After puncture obtained the tract was dilated with serial Amplatz’s dilators, the stones were crushed with pneumatic lithotripsy and removed completely (Fig. 4A and B), followed by removal of the encrusted stent at the end (Fig. 5).

**Figure 6:**
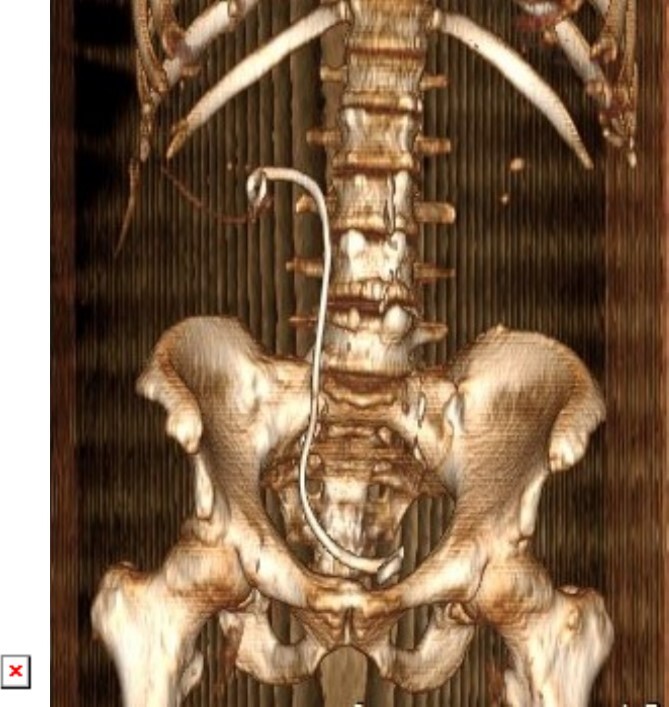
3D constructed image of postoperative low dose non-contrast CT scan showing stone clearance and newly inserted DJ stent in-situ.

Postoperatively, a low dose non-contrast CT scan confirmed stone clearance (Fig. 6). Subsequent outpatient follow-up with renal ultrasound showed no residual hydronephrosis. The newly inserted double J stent was removed three weeks after the percutaneous nephrolithotomy procedure.

## Discussion

Ureteral stents play a vital role in urological procedures, but stent encrustation, characterized by mineral build-up on the stent’s surface, poses challenges due to associated complications like infection, obstruction, and fragmentation [4]. Factors contributing to encrustation include patient-specific issues like recurrent infections, stone formation tendencies, and metabolic abnormalities [5]. Stent material and surface characteristics also play a role [6].

Prolonged stent placement is a significant risk factor for encrustation [3]. In our case, the stent was neglected for 18 years due to the patient’s military duties and low health knowledge, leading to extensive encrustation. Poor patient compliance and health literacy increase the likelihood of retained stents. [3].

Various grading systems help assess the severity of encrustation and predict the challenges of stent removal. The FECal system categorizes encrustation based on size, location, and degree of encrustation [7], while the KUB system grades different parts of the stent individually based on encrustation extent [8]. An alternative treatment approach proposed by Nir tomer, Evan Garden, and their colleagues integrates these systems, suggesting cystoscopic removal for mild encrustation and surgical intervention for severe cases [4].

In cases like ours with significant encrustation, advanced multi-modal approaches like PCNL and open surgery are often necessary for successful removal of stones and stents. This combined approach resulted in a successful outcome for our patient.

## Conclusion

The issue of stent encrustation in urology is a significant concern that affects patient outcomes, requiring a comprehensive approach for management. Strategies such as optimizing stent duration, using stents with anti-encrustation properties, and implementing regular monitoring protocols are crucial to prevent serious long-term complications. Further research on biodegradable stents is warranted as they may offer cost benefits by eliminating the need for additional treatments associated with forgotten and retained stents, especially in resource-limited settings [9]. Proposals like a national stent registry, reminder systems, and improved communication between healthcare providers and patients aim to reduce the incidence of forgotten stents and subsequent encrustation [10].

Given the complexities associated with forgotten and encrusted DJ stents, proactive measures are essential. Stents should be promptly removed when no longer necessary and replaced periodically if required for extended periods.
